# TRPM2 and warmth sensation

**DOI:** 10.1007/s00424-018-2139-7

**Published:** 2018-03-19

**Authors:** Chun-Hsiang Tan, Peter A. McNaughton

**Affiliations:** 1 Department of Neurology, Kaohsiung Medical University Hospital, Kaohsiung Medical University, Kaohsiung, Taiwan; 2 Graduate Institute of Clinical Medicine, College of Medicine, Kaohsiung Medical University, Kaohsiung, Taiwan; 3 Wolfson Centre for Age-Related Diseases, Institute of Psychiatry, Psychology and Neuroscience, Guy’s Campus, King’s College London, London, SE1 1UL UK

**Keywords:** Thermal sensation, Warmth, Heat, Ion channel, TRP, TRPM2, Sex differences, Autonomic nervous system

## Abstract

The abilities to detect warmth and heat are critical for the survival of all animals, both in order to be able to identify suitable thermal environments for the many different activities essential for life and to avoid damage caused by extremes of temperature. Several ion channels belonging to the TRP family are activated by non-noxious warmth or by heat and are therefore plausible candidates for thermal detectors, but identifying those that actually regulate warmth and heat detection in intact animals has proven problematic. TRPM2 has recently emerged as a likely candidate for the detector of non-noxious warmth, as it is expressed in sensory neurons, and mice show deficits in the detection of warmth when TRPM2 is genetically deleted. TRPM2 is a *chanzyme*, containing a thermally activated TRP ion channel domain attached to a C-terminal motif, derived from a mitochondrial ADP ribose pyrophosphatase, that confers on the channel sensitivity to ADP ribose and reactive oxygen species such as hydrogen peroxide. Several open questions remain. Male mammals prefer cooler environments than female, but the molecular basis of this sex difference is unknown. TRPM2 plays a role in regulating body temperature, but are other warmth-detecting mechanisms also involved? TRPM2 is expressed in autonomic neurons, but does it confer a sensory function in addition to the well-known motor functions of autonomic neurons? TRPM2 is thought to play important roles in the immune system, in pain and in insulin secretion, but the mechanisms are unclear. TRPM2 has to date received less attention than many other members of the TRP family but is rapidly assuming importance both in normal physiology and as a key target in disease pathology.

## Introduction

In order to survive, all animals must detect environmental temperatures and react appropriately to them. Extreme temperatures can cause damage, and even subtle changes in the environmental temperature can cause changes in the physiology of living organisms. Animals must therefore be capable of detecting noxious thermal extremes, to avoid injury from extremes of heat or cold, and must also be equipped with the ability to detect optimal environmental temperatures in which to carry out different activities such as hunting, feeding, resting, and reproducing.

Thermal stimuli can be broadly categorized into painful heat, innocuous warmth, innocuous coolness, and painful cold. The mean detection thresholds for innocuous warmth and innocuous coolness range between 1.3 to 6.2 °C above and 0.9 to 3.4 °C below the baseline skin temperature, respectively. The mean detection threshold for painful heat is between 41.5 and 47.0 °C and for painful cold is more variable, at between 7.3 and 18.4 °C [95]. Differences in age, in the anatomical region tested, in ethnicity, and in sex have all been shown to influence the thermal detection thresholds [20, 26, 66, 67, 95]. Generally, the older the age, the higher the thresholds for detection of thermal stimuli, while face and hands are usually more sensitive [67, 95]. Ethnicity differences in thermal detection and thermal pain between Chinese and Danes have been reported, with people of Chinese origin being more sensitive to thermal stimuli [132]. Sex differences in response to thermal stimuli are most prominent for thermal pain thresholds, with females being more sensitive to both painful heat and painful cold [95, 121]. Although most studies did not show a statistically significant difference between sexes in the thresholds for thermal *detection* of innocuous warmth or coolness, several studies have shown a sex difference in thermal *preference*. The preferred environmental temperature for human males, 22 °C, is significantly lower than that for females (25 °C) [5, 48]. Moreover, one study found that females are more likely to show thermal discomfort in cool environments while males to be more likely to feel uncomfortable in warm environments [128]. In addition, women have a higher mean core temperature and a lower mean skin temperature than men [52].

The cellular mechanisms of thermosensation were initially examined by recording the activation of afferent nerve fibers in response to thermal stimuli in the glossopharyngeal nerve and the chorda tympani of cats [135]. Studies with lingual nerves also showed that both cold and warm receptors existed [136], and that receptor activation elicited steady discharges of impulses at constant temperatures, with maximum frequencies observed between 25 and 35 °C for cold receptors and 37.5 and 40 °C for warm receptors [19, 37]. Discharges elicited by thermal stimuli were shown in later studies in afferents from the cat’s skin [35, 36, 44]. Based on the conduction velocity of the electrical discharges, the transmission of the thermal stimuli signal was found to rely on unmyelinated C fibers or thinly myelinated Aδ fibers [16, 35, 44]. The detection of skin temperature in subprimates and primates was also shown to depend on cutaneous thermoreceptors and the propagation of action potentials in slowly conducting nerve fibers [16, 35, 44].

## TRP ion channels activated by warmth and heat

Although the existence of thermoreceptors was demonstrated more than 80 years ago in the studies mentioned above, the molecular mechanisms underlying the detection of skin temperature by thermoreceptors remained elusive until recently, when noxious heat stimuli were shown to rapidly elicit an inward current in a subpopulation of small primary sensory neurons [11]. The reversal potential of the heat-activated ion current was very close to 0 mV, showing that the heat-activated ion channel discriminates poorly among cations. One of the heat-activated ion channels responsible for the heat sensitivity of primary sensory neurons was subsequently cloned by adopting a strategy based on calcium imaging of HEK293 cells expressing cDNA isolated from sensory neurons, and using capsaicin, the “hot” ingredient of chili peppers, to isolate a functional cDNA encoding a capsaicin receptor [10]. Capsaicin is a member of the chemical family named the vanilloids, and the channel was at first called vanilloid receptor subtype 1 (VR1) [10], though to recognize its homology with other members of the transient receptor potential (TRP) ion channel family, it has now been renamed transient receptor potential vanilloid 1 (TRPV1) under the unified nomenclature for the family of TRP cation channels [15]. Several other TRP channels activated by warmth or heat were identified soon after the cloning of TRPV1. The definition of thermosensitivity is based on the temperature coefficient (Q_10_) value, which is calculated as the relative increase in current amplitude when the temperature increases by 10 °C. Heat-sensitive TRP channels are generally defined as TRP channels with Q_10_ values higher than 5, and the thermally sensitive TRP channels have been identified on this criterion as TRPV1, TRPV2, TRPV3, TRPV4, TRPM2, TRPM3, TRPM4, and TRPM5 [123].

TRPV1 has a Q_10_ value of 25, an activation threshold at temperature > 42 °C, and carries a nonselective cation current displaying outward rectification [122]. In addition to heat stimuli and capsaicin, a wide range of other agonists, including resiniferatoxin [97], ethanol [116], anandamide [99], and extracellular protons [113] also activate TRPV1. Both C and Aδ nociceptive fibers of somatosensory neurons express TRPV1, and many TRPV1-positive fibers also co-express the proinflammatory neuropeptides substance P, neurokinin A, and calcitonin gene-related peptide (CGRP), substantiating a proinflammatory role for TRPV1 [46, 56, 113]. Shortly after the cloning of TRPV1, the same group also successfully cloned a TRPV1 homolog, subsequently named TRPV2 [9]. TRPV2 has a Q_10_ value higher than 100, with an even higher activation threshold, at 52 °C, than TRPV1. Based on its thermal sensitivity, TRPV2 was initially proposed as a candidate for a high-temperature, TRPV1-independent mechanism of heat sensation [9]. Like TRPV1, the activation of TRPV2 elicits a nonselective cation current displaying outward rectification [15, 63]. In addition to heat, hypo-osmolarity, membrane stretch, and cannabinoids also activate TRPV2 [77, 93]. One notable difference from TRPV1 is that TRPV2 is expressed in medium- to large-diameter myelinated sensory neurons, and in addition to sensory neurons, TRPV2 is also expressed in the epithelium of several different tissues (including pancreatic duct, mammary gland, parotid gland, submandibular gland, renal tubule, and tracheal gland) and in immune cells [59, 65].

TRPV3, also a close homolog of TRPV1, has a Q_10_ value higher than 6 and an activation threshold at temperatures around 31 °C, and carries an outwardly rectifying nonselective cation current [15, 86, 100, 130]. Carvacrol, eugenol, and thymol, found in plants such as oregano, savory, clove, and thyme, are agonists for TRPV3 [129]. TRPV4 was cloned as an osmotically sensitive channel carrying an outwardly rectifying nonselective cation current [14, 64, 105] and was found to be thermally sensitive almost 2 years after cloning [29]. The activation threshold of TRPV4 by non-noxious heat is around 25 °C [14] with a Q_10_ value of around 10 [29]. In addition to hypotonic stress and non-noxious heat, bisandrographolide from the Chinese herbal plant *Andrographis paniculata* can also activate TRPV4 [101]. Both TRPV3 and TRPV4 are expressed in skin keratinocytes, but not in neurons, and have therefore been proposed to act as non-noxious warmth sensors whose excitation could be transferred to primary afferent terminals of sensory neurons by a factor released from keratinocytes [13, 68, 86].

TRPM2 was cloned in 1998 with an initial goal of finding genes responsible for several diseases such as autoimmune polyglandular disease type I, bipolar affective disorder, and nonsyndromic hereditary deafness [78]. TRPM2 carries a voltage-insensitive nonselective cation current with a linear current-voltage relation. The thermosensitivity of TRPM2 was not discovered until 2006, when it was demonstrated that TRPM2 can be activated upon exposure to a temperature above 35 °C, with a Q_10_ value at around 15.6 [112]. In addition to heat, the activity of TRPM2 can be synergistically modulated by intracellular ADP-ribose (ADPR) and Ca^2+^ [34] and can also be enhanced by additional factors, such as cyclic ADP-ribose and nicotinic acid adenine dinucleotide phosphate (NAADP) [4, 58]. Reactive oxygen species (ROS), such as H_2_O_2_, can also cause activation or sensitization of TRPM2 [30, 49, 126]. TRPM3, another member of the TRPM subfamily, carries an outwardly rectifying nonselective cation current and has an activation threshold at a temperature above 40 °C, with a Q_10_ value of 7.2 [124]. Pregnenolone sulphate, nifedipine, and β-cyclodextrin have been shown to activate TRPM3 [79, 125]. TRPM3 is abundantly expressed in small diameter dorsal root ganglion neurons and also in a variety of other tissues, including kidney, liver, ovary, brain, spinal cord, pituitary, vascular smooth muscle, and testis [27, 62, 79, 124].

Unlike most of the other members of the TRPM subfamily, which are nonselective cationic channels, TRPM4 and TRPM5 are not permeable to divalent cations [28]. TRPM4 carries an outwardly rectifying current that has a Q_10_ value of around 8.5 between 15 and 25 °C [108]. Intracellular calcium, decavanadate and BTP2 are also activators of TRPM4, in addition to heat [80, 107, 120]. TRPM4 is widely expressed in a variety of tissues, with high expression in the heart, pancreas, placenta, and prostate, and lower levels in the kidney, skeletal muscle, liver, intestines, thymus, and spleen in humans [1]. TRPM5 carries an outwardly rectifying current that has a Q_10_ value of around 10.3 with a threshold between 15 and 25 °C. Like TRPM4, TRPM5 can also be activated directly by intracellular calcium, and the concentration of intracellular calcium leading to activation of TRPM5 is even lower than that of TRPM4, though higher concentrations of intracellular calcium cause inhibition of TRPM5 [92, 120].

## Roles of TRP channels as physiological sensors of warmth and heat

Although all the heat-sensitive TRP channels mentioned above display Q_10_ values higher than 5 in vitro, and therefore are plausible thermal sensors covering the entire range over which thermal detection is known in take place in animals, attempts to identify the thermosensors that are actually responsible for the physiological detection of warmth and heat in intact animals have not in general met with great success. TRPV1 knockout mice show a partial deficit in sensing noxious heat, but they still withdraw their tails from hot water [8], and, more importantly, no significant difference was noted between TRPV1 knockout mice and wild-type mice when evaluated with the two-plate thermal preference test [91]. Although TRPV2 was at first suggested to be responsible for the TRPV1-independent mechanism of heat sensation, behavioral assays of TRPV1/TRPV2 double knockout mice, or TRPV2 knockout mice treated with resiniferatoxin to desensitize TRPV1-expressing afferents, reveal no thermosensory deficits resulting from genetic deletion of TRPV2 [83]. Genetic deletion of TRPV3 in mice on an intercrossed C57BL6/129J background was reported initially to cause relatively minor deficits in responses to innocuous and noxious heat stimuli [75]. However, TRPV3 knockout mice on a homogeneous C57BL6 background exhibited no obvious changes in thermal preference behavior, while deletion of TRPV3 in a 129S6 background resulted in a more restricted range of occupancy centered around cooler floor temperatures [43]. Meanwhile, TRPV3 knockout mice showed no deficits in acute heat nociception on either a C57BL6 or a 129S6 background [43]. Importantly, although the activation threshold for TRPV3 and TRPV4 by heat is in the range of innocuous warmth, mice deficient in both TRPV3 and TRPV4 show a thermal preference behavior on a thermal gradient similar to wild-type mice and little or no change in acute heat perception [43]. These results demonstrate that TRPV3 and TRPV4 do not play important roles in thermosensation and that their contribution to thermosensation, if any, can be strongly influenced by genetic background. Mice with a genetic deletion of TRPM3 exhibited reduced but not absent sensitivity to noxious heat, in a similar manner to TRPV1 knockout mice. Although during the exploration period TRPM3 knockout mice spend significantly more time at temperatures between 31 and 45 °C than wild-type mice, the thermal preference behavior of wild-type and TRPM3 knockout mice was very similar on a surface temperature gradient of 5 to 60 °C [124]. Regarding the other two warmth-sensitive TRP channels, TRPM4 and TRPM5, no studies investigating the functions of the two channels in thermosensation have been reported.

In contrast to the lack of a clear role for any individual TRP channel in the sensation of warmth or heat, the TRPM8 ion channel, which is activated by cool temperatures [70, 85], plays a clear role in determining the thermal preference of mice over the temperature range of non-noxious coolness between 15 and 30 °C [2, 17, 54, 55]. Thus, the only thermally activated TRP ion channel, out of those discussed above, that unambiguously confers thermal sensitivity upon the behavior of an intact animal appears to be the “cool” receptor, TRPM8.

## Molecular basis of warmth sensation

The molecular mechanisms underlying non-noxious warmth sensation remained elusive until we recently demonstrated an essential role for TRPM2 [109]. As noted above, mouse models in which TRPV1, TRPV2, TRPV3, TRPV4, and TRPM3 had been genetically deleted showed no clear deficits in thermal sensation in the range of innocuous warmth between 30 and 40 °C. In addition, the fact that nearly 50% of dorsal root ganglion (DRG) neurons from TRPM3 KO mice still responded to heat, even when TRPV1 is blocked by a potent and selective TRPV1 antagonist, suggests the presence in sensory neurons of an additional thermal sensor or sensors other than TRPM3 or TRPV1 [124]. We set out to discover novel heat-sensitive mechanisms using calcium imaging. Our strategy was first to identify somatosensory neurons expressing TRPV1, TRPV2, TRPV3, TRPV4, or TRPM3, using agonists of these known thermo-TRP channels, and then to focus on the properties of the novel thermally activated neurons that did not express any of these known thermo-TRPs. We found that around 10% of DRG neurons were heat-activated but did not express any known thermo-TRP, and therefore express the proposed novel heat-sensitive mechanism. This finding does not necessarily indicate that the expression of the novel heat-sensitive mechanism is limited to the 10% of DRG neurons, as it is possible that the novel heat-sensitive mechanism could also be co-expressed with other thermo-TRP channels. We therefore tested the responses of DRG neurons to heat stimuli after blocking TRPV1 with AMG9810 and TRPM3 with naringenin, and we found that 46% of DRG neurons responded to heat, suggesting significant co-expression of the novel heat-sensitive mechanism with TRPV1 and TRPM3. Around half of DRG neurons therefore express the novel heat-sensitive mechanism, with around 10% expressing it in isolation, and a further ~ 40% in combination with TRPV1 and/or TRPM3.

In order to better characterize the novel heat-sensitive mechanism, we sought to identify a source of neurons expressing a less complex set of heat-sensitive ion channels than is present in DRG neurons. In autonomic ganglion neurons, both sympathetic and parasympathetic, we found that around half of isolated neurons showed a significant calcium increase in response to heat, but that very few neurons showed any response to agonists of known thermo-TRP channels. Autonomic neurons therefore express the novel heat-sensitive mechanism in isolation, which offers an advantageous preparation for characterizing its properties. Heat-evoked increases of the intracellular calcium concentration were completely abolished by removal of extracellular calcium, and so were caused by an influx of calcium from the extracellular solution. In addition, the calcium influx was reduced but not abolished in the absence of extracellular sodium or in the presence of L-type calcium channel blockers, and was unaffected by the voltage-dependent sodium channel blocker tetrodotoxin or by the TRPV channel blocker ruthenium red. These results indicated that the heat-evoked calcium entry occurred through a mixed sodium and calcium-permeable channel, and that in the presence of sodium, the main current carrier, depolarization of neurons to the threshold for generation of action potentials by voltage-gated calcium channels caused an additional calcium influx. By evaluating the current-voltage relations of autonomic neurons voltage-clamped in the whole-cell configuration at 36 and 47 °C, with all conventional voltage-dependent ion channels blocked, the current-voltage relation of the heat-activated ion channel was shown to be approximately linear, with a reversal potential close to 0 mV. The properties of mixed sodium and calcium permeability and a linear current-voltage relation with a reversal potential close to 0 mV suggested the channel to be a TRP ion channel, or possibly a member of the cyclic-nucleotide-gated (CNG) ion channel family.

To determine the molecular identity of the novel heat-sensitive ion channel, we carried out an analysis of RNA sequencing on a sympathetically derived cell line, MAH cells, which are similar to sympathetic neurons in their thermal responses, in that there are no responses to agonists of known thermo-TRP channels, but the novel heat-sensitive mechanism is present in a significant proportion of cells. When differentiated by a cocktail of growth factors, the heat sensitivity of the MAH cells was found to be reduced. RNA sequencing showed that the only channels expressed in MAH cells, out of all TRP and CNG channels, were TRPC1, C2, C3, V2, M2, M4, and M7. Among these, only TRPM2 has a linear current-voltage relation, a mixed sodium and calcium permeability, and was not upregulated by differentiation. The molecular identity of the novel heat-sensitive ion channel was confirmed in sensory and sympathetic neurons from TRPM2^−/−^ mice, in which the percentage of novel heat-sensitive neurons, and the amplitudes of the residual heat responses in the few remaining heat-responsive neurons, were both greatly reduced [109].

## Properties of TRPM2

TRPM2 is a fascinating example of a *chanzyme*, a membrane protein containing both a functional ion channel and an enzymatic domain. The intracellular C terminus contains a nudix hydrolase (NUDT9) homology region, which was named after the mitochondrial ADP-ribose (ADPR) pyrophosphatase NUDT9 and gives TRPM2 the chanzyme designation, although the NUDT9 domain in TRPM2 does not appear to be functional in the enzymatic sense and the channel is activated simply by binding ADPR [45, 72, 74, 87, 98, 115]. The full-length long form TRPM2 protein in humans consists of 1503 amino acid residues (1506 in mouse and 1507 in rat) and is encoded by 32 exons [78, 104]. In addition to the nudix hydrolase domain, the C-terminal contains a coiled-coil region governing channel assembly [72]. The N terminus of the full-length TRPM2 contains four domains of the TRPM homology region, including a calmodulin-binding IQ-like motif providing positive feedback for channel activation, followed by six transmembrane domains with a pore-forming loop domain between S5 and S6 [71, 87, 114]. Partial proteolysis of mitochondrial NUDT9 suggests that NUDT9 enzymes consist of two domains, a C-terminal CORE domain containing segments with ADPR pyrophosphatase activity and an N-terminal CAP domain with the function of enhancing the affinity of the C-terminal CORE domain for ADPR [89]. The nudix hydrolase homology domain (NUDT9-H) was shown to be of importance for normal channel assembly and surface trafficking, because deletion of the domain significantly decreases the membrane expression of TRPM2 [90].

In addition, a variety of splice variants of TRPM2 have been reported. The first splice variant, cloned from HL-60 cells, is characterized by deletions in the N-terminal region (nucleotides 2056–2115) and in the C-terminal region (nucleotides 4318–4419), and is denoted as TRPM2-ΔNΔC. TRPM2-ΔN was shown to be a dysfunctional splice variant of TRPM2, as evidenced by the finding that the sensitivity to H_2_O_2_ and ADPR was abolished in HEK293 cells transfected with cDNA of the splice variant [126]. Although the deleted segment overlaps with two IQ-like calmodulin-binding domains and contains two PxxP motifs implicated in protein–protein interactions, the dysfunction was also shown to be unrelated to any of these motifs as removal of each of the two IQ-motifs or deletion of either one or both PxxP motifs caused no functional loss. The findings suggest that the ΔN-stretch may be a spacer segment for other functional sites [60]. TRPM2-ΔC was shown to be unresponsive to ADP-ribose [126]. However, the responses of the splice variant to H_2_O_2_ varies among different studies, with some studies showing that TRPM2-ΔC could be activated by H_2_O_2_ as efficiently as the wild type [81, 126], while another study shows the ΔC splice variant to be unresponsive to H_2_O_2_ [90]. Whether H_2_O_2_ directly activates TRPM2, or whether its effect occurs indirectly via a change in ADPR, is therefore uncertain. The studies showing activation of TRPM2-ΔC by H_2_O_2_ suggest that the deletion mutant is expressed on the surface membrane, and moreover that the action of H_2_O_2_ does not depend on ADPR as mediator [81, 126]. Results showing that deletion of the NUDT9-H domain significantly decreases membrane expression [90] while deletion of the amino acids in the NUDT9-H CAP region of the TRPM2-ΔC channels preserves the membrane expression of TRPM2 [88], suggest that the NUDT9-H CORE domain is involved in the functional expression of TRPM2 on the membrane. The striatal short form (TRPM2-SSF) lacks 214 N-terminal amino acid residues but has intact H_2_O_2_-induced Ca^2+^ influx activity [119]. TRPM2-AS is a short form of TRPM2 consisting of the TRPM2 N terminus and the first two predicted transmembrane domains, resulting from alternative splicing which locates a stop codon (TAG) at the splice junction between exons 16 and 17 [133]. Owing to the lack of the pore region and the C terminus, the splice variant exerts its function in a dominant negative form and has been reported to inhibit TRPM2-mediated calcium influx and thus to reduce susceptibility to cell death caused by exposure to H_2_O_2_ [133, 134]. Consistent with this, the short form splice variant was also shown by co-immunoprecipitation to interact with the long form TRPM2 and can exert inhibition of the long form TRPM2 [133]. Although heteromers composed of various combinations of TRP channels have been reported [21, 57], whether TRPM2 interacts with other TRP channels through heteromerization remains unknown.

The activation of TRPM2 by warmth was first shown in HEK293T cells heterologously expressing TRPM2, and the amplitude of the TRPM2 current evoked by ADPR was also shown to be enhanced at elevated temperatures [112]. Beta-NAD^+^- and cyclic-ADP-ribose evoke little TRPM2 current at 25 °C, but a temperature of 40 °C greatly enhances the currents evoked by beta-NAD^+^- and cyclic-ADP-ribose [112]. Although the *amplitude* of TRPM2 current can be enhanced in the presence of beta-NAD^+^- and cyclic-ADP-ribose, the *temperature threshold* of TRPM2 was reported to be unaffected by the presence of beta-NAD^+^- or cyclic-ADP-ribose [112]. In contrast, H_2_O_2_ appears to sensitize the heat responses mediated by TRPM2 by lowering the temperature threshold of activation to the range around body temperature both in a transient expression system and in macrophages [49]. The thermosensitivity of TRPM2 and the effect of H_2_O_2_ in sensitizing the thermal responses of TRPM2 were also demonstrated in sensory and autonomic neurons [109].

TRPM2 is widely expressed in the central and peripheral nervous system [23], both in nonneuronal microglial cells and in neurons [12, 22]. In addition, a relatively high level of TRPM2 expression was shown in a variety of immune cells [23], including neutrophils [32–34, 38, 61, 84], monocytes [87, 127, 131], macrophages [49, 137], dendritic cells [84, 106], mast cell [82], and lymphocytes [4, 6, 94]. The expression pattern of TRPM2 and its sensitivity to temperatures in the range of mammalian body temperature suggest a potential role in thermoregulation, in the immune system, and in inflammatory responses.

## TRPM2 is responsible for the detection of non-noxious warmth

In view of the expression of TRPM2 in novel heat-sensitive neurons, we investigated whether TRPM2 might be responsible for non-noxious warmth preference [109]. The thermal preference of wild-type adult male mice for a temperature around 33 °C has been well documented with the two-plate thermal preference test in a number of studies [43, 73, 91]. We used this system with one control plate at 33 °C, the preferred temperature, while the temperature of the test plate was adjusted to cooler or warmer temperatures. The test and control plates were reversed after 30 min to control for any influence of environmental cues. The results showed that adult male wild-type mice avoided the non-noxious warm temperature of 38 °C, in agreement with the results previously published [43, 73, 91], while TRPM2 knockout mice showed no avoidance of 38 °C [109]. Overall, deletion of TRPM2 caused mice to prefer warmer temperatures over a wide thermal range from 23 to above 38 °C, suggesting that TRPM2 is activated by warmth and creates an aversive signal that drives male mice towards cooler temperatures [109]. Thermal behavior may therefore be thought of as a balance between an aversive “warm” signal, created by TRPM2, and an aversive “cool” signal, created by TRPM8 as discussed above [2, 17, 54, 55], with the optimal environmental temperature achieved when the two signals are in balance.

## Sex differences in thermal preference

In adult wild-type male mice, a preference for 33 °C over cooler or warmer floor temperatures has been well documented [43, 73, 91], but female mice have a distinct preference for warmer temperatures [47, 73]. Altered female behaviors in response to different ambient temperatures, usually a slightly warmer and less prominent thermal preference than male mice, were also observed [24, 25]. In humans, women prefer a warmer ambient temperature than men [5, 48] and also on average have a cooler peripheral skin temperature than men, along with a warmer core temperature [52]. These differences are therefore consistent across labs, paradigms, and species, but the reasons for the influence of gender on thermal behavior remains both interesting and unknown. Is the difference driven by sex hormones or by other genetic factors? Is it due to a difference in peripheral thermal perception, perhaps driven by sex differences in TRPM2 and/or TRPM8, or is it attributable to other factors?

## Participation of TRPM2 in central thermal sensation

In view of the role of TRPM2 in sensing ambient warm temperatures, a possible role for TRPM2 is attractive in the sensation of, and perhaps also in the control of, normal body warmth. TRPM2 was found to be expressed in a subpopulation of neurons in the preoptic area of the hypothalamus, which is known to play an important role in the sensation of central temperatures and in whole-body thermoregulation [103]. In calcium imaging experiments, these neurons were found not to be active at the normal body temperature of 37 °C, in both cell cultures and in brain slices, but to be activated by small temperature elevations, to 38°C or above, in brain slices. Consistent with this, normal body temperature was unaffected by genetic deletion of TRPM2, and mild fever induced by injection of a small dose of PGE_2_ into the hypothalamus was also unaffected. However, a larger increase in body temperature was noted in TRPM2 knockout mice after injecting a higher dose of PGE_2_ or other pyrogenic stimuli into the hypothalamus, suggesting that TRPM2 plays a role as a “brake” which prevents the development of an excessive fever response. Chemogenetic activation and inhibition of hypothalamic TRPM2-expressing neurons in vivo caused a decrease and increase of body temperature, respectively, showing that these neurons are potently connected to neural circuits involved in the control of body temperature [103]. Overall, this fascinating work suggests that TRPM2 does not play a central role in the maintenance of normal body temperature nor in mild fever but appears instead to act as an “emergency response system” which is able to bring into play potent thermoregulatory responses in order to reduce body temperature when it rises to dangerous levels.

## Role of TRPM2 in the autonomic nervous system

Neurons of the autonomic nervous system, both sympathetic and parasympathetic, express a warm-sensory function mediated by TRPM2, as described above [109]. This finding is surprising because the autonomic nervous system has been traditionally viewed as carrying out a purely motor function, and the idea that it may have an autonomous sensory function has not, to the best of our knowledge, been considered before.

The prevailing concept of thermoregulation holds that the peripheral somatosensory system detects the external environment temperature and sends information to the hypothalamus, where it is integrated with information on core body temperature detected in the preoptic area. The sympathetic nervous system then serves as the output pathway that transmits the integrated thermal command to control the vasomotor tone of surface blood vessels, the activity of brown adipose tissue, and sweating [76]. However, the model ignores a possible effect of temperature acting directly and locally on tissues. For example, local warming has long been known to cause local vasodilation [110], an effect that has been attributed to a reflex release of vasodilator peptides such as CGRP from activated sensory nerve endings. However, one study showed that when conduction of action potentials in sensory nerves was blocked with anesthetic cream, the steady-state vasodilation by local warming up to 42 °C was not different from control, but that the vasodilation was significantly reduced following inhibition of exocytosis from postganglionic sympathetic neurons with bretylium [7]. This result suggests that the peripheral sympathetic nervous system is more important than the somatosensory nervous system for the steady-state vasodilation evoked by local warming. It may be counter-intuitive to believe that postganglionic sympathetic neurons have an important thermosensory role in the vasodilation evoked by local warming, but evidence for this view is growing because the inhibition of local warming-evoked vasodilation by bretylium has now been shown in many studies [39, 40, 42, 111]. It would be of great interest to investigate a potential physiological role for TRPM2 as a thermosensor in the autonomic nervous system.

## Role of TRPM2 in the immune system

TRPM2 is widely expressed in the immune system [96], and the sensitivity of TRPM2 to oxidative stress suggests a potential involvement of TRPM2 in inflammatory responses [30, 126]. The oxidative stress-induced Ca^2+^ influx through TRPM2 initiates downstream reactions essential for chemokine production and the resultant inflammatory responses. The crucial role of TRPM2 in inflammatory responses is supported by the impairment of H_2_O_2_-induced production of macrophage inflammatory protein-2 (MIP2, also known as CXCL2) in monocytes from TRPM2 knockout mice [131]. TRPM2 was shown to participate in the enhancement of bactericidal activity by lysophosphatidylcholine (LPC) via a signaling pathway involving glycine receptor α2, TRPM2, and p38 mitogen-activated protein kinases [41]. Furthermore, TRPM2 knockout mice were shown to be highly susceptible to infection with *Listeria monocytogenes* (*Lm*) and exhibited impaired innate immunity with reduced level of cytokines IL-12 and IFNγ after *Lm* infection [53]. These studies suggest that TRPM2 plays an *enhancing* role in inflammatory responses.

On the other hand, one study evaluated the function of TRPM2 in a model of endotoxin-induced lung inflammation and found increased release of chemokines and proinflammatory cytokines, including tumor necrosis factor, the chemokine CXCL2 and interleukin 6, in the lungs of TRPM2 knockout mice. These results suggest that TRPM2 plays a negative feedback role in dampening the inflammatory responses induced by oxidative stress [18]. Another study showed that TRPM2 knockout mice chronically infected with *Helicobacter pylori* exhibited increased gastric inflammation and greater macrophage production of inflammatory mediators and macrophage M1 polarization. These results, in contrast, are in favor of an *inhibitory* role of TRPM2 in inflammatory responses [3]. The exact role of TRPM2 in inflammatory responses warrants further clarification.

## Role of TRPM2 in pain

A role for TRPM2 in the pathogenesis of inflammatory and neuropathic pain is suggested from the expression of TRPM2 both in the peripheral nervous system and in the immune system, together with the fact that TRPM2 is activated by oxidative stress. One study showed that in carrageenan-induced inflammatory pain and sciatic nerve injury-induced neuropathic pain models, mechanical allodynia and thermal hyperalgesia were attenuated in TRPM2 knockout mice [31]. Another study showed that the severity of pathological pain conditions are significantly reduced in TRPM2 knockout mice; the models investigated include acetic acid-induced writhing behavior, mechanical allodynia in the monosodium iodoacetate-induced osteoarthritis pain model, the experimental autoimmune encephalomyelitis model, the paclitaxel-induced peripheral neuropathy model, and the streptozotocin-induced painful diabetic neuropathy model [102]. Furthermore, the trinitrobenzene sulfonic acid-induced visceral hypersensitivity was significantly reduced in TRPM2 knockout mice, while the enhanced visceromotor response to noxious colorectal distention induced in colitis model was restored to the control level after treatment with econazole, a TRPM2 inhibitor [69]. These results show that TRPM2 promotes the development of pathological pain states. Selective blockers of TRPM2 may find a future use as analgesics.

## Role of TRPM2 in insulin secretion

TRPM2 is expressed in pancreatic beta cells, which under normal circumstances are not exposed to significant changes in temperature. Reactive oxygen species such as H_2_O_2_ could, however, modulate TRPM2 and therefore influence the secretion of insulin. In isolated pancreatic islet cells, H_2_O_2_ induces both a greater calcium increase and enhanced insulin secretion when compared to its effect in cells from TRPM2 knockout mice [50, 117]. A physiological role for TRPM2 is suggested from the observations that TRPM2 knockout mice show attenuated insulin secretion and higher blood glucose levels in glucose tolerance tests [51, 118]. TRPM2 may therefore be an important participant in the physiology of glucose homeostasis, though the details of the mechanism are yet to be fully elucidated.

## Conclusion

Over the last decade, we have gained a much deeper understanding of the role of TRPM2 in thermosensation and thermoregulation, in the immune system and in pain, but there are still many unresolved questions. Why is thermosensation sex-dependent, and is the difference driven by TRPM2? Does TRPM2 contribute to the crucial mechanism that maintains basal core body temperature at around 37 °C? Present evidence suggests that it does not—but is TRPM2 instead involved in initiating or regulating fever? TRPM2 appears to be implicated in the responses of the immune system, which are known to be strongly temperature-dependent, but the mechanistic details are unclear. There are conflicting results regarding the role of TRPM2 in inflammatory responses. All these questions are of crucial importance and need further investigation.
